# National intensive care unit bed capacity and ICU patient characteristics in a low income country

**DOI:** 10.1186/1756-0500-5-475

**Published:** 2012-09-01

**Authors:** Arthur Kwizera, Martin Dünser, Jane Nakibuuka

**Affiliations:** 1 Department of Anaesthesia, Anaesthesia and General Intensive Care, Mulago Hospital and Makerere University, Mulago Hill Road, Kampala, Uganda; 2 Department of Anaesthesiology, Perioperative Medicine and General Intensive Care, Salzburg General Hospital and Paracelsus Private Medical University, Salzburg, 5020, Austria; 3 Department of Medicine, Internal Medicine and General Intensive Care, Mulago Hospital and Makerere University, Mulago Hill Road, Kampala, Uganda

**Keywords:** Intensive care medicine, Diagnosis, Uganda, Low-income country, Mortality

## Abstract

**Background:**

Primary health care delivery in the developing world faces many challenges. Priority setting favours HIV, TB and malaria interventions. Little is known about the challenges faced in this setting with regard to critical care medicine. The aim of this study was to analyse and categorise the diagnosis and outcomes of 1,774 patients admitted to a hospital intensive care unit (ICU) in a low-income country over a 7-year period. We also assessed the country’s ICU bed capacity and described the challenges faced in dealing with critically ill patients in this setting.

**Findings:**

A retrospective audit was conducted in a general ICU in a university hospital in Uganda. Demographic data, admission diagnosis, and ICU length of stay were recorded for the 1,774 patients who presented to the ICU in the period January 2003 to December 2009. Their mean age was 35.5 years. Males accounted for 56.5% of the study population; 92.8% were indigenous, and 42.9% were referrals from upcountry units. The average mortality rate over the study period was 40.1% (n = 715). The highest mortality rate (44%) was recorded in 2004 and the lowest (33.2%) in 2005. Children accounted for 11.6% of admissions (40.1% mortality). Sepsis, ARDS, traumatic brain injuries and HIV related conditions were the most frequent admission diagnoses. A telephonic survey determined that there are 33 adult ICU beds in the whole country.

**Conclusions:**

Mortality was 40.1%, with sepsis, head injury, acute lung injury and HIV/AIDS the most common admission diagnoses. The country has a very low ICU bed capacity. Prioritising infectious diseases poses a challenge to ensuring that critical care is an essential part of the health care package in Uganda.

## Findings

### Background and research hypothesis

The prevalence of critical illness in developing countries is disproportionately high in view of the disproportionate burden of diseases such as HIV/AIDS, malaria, tuberculosis and trauma. Sub-Saharan Africa bears 25% of the global burden of disease [1]. Management of critically ill patients requires significant human, infrastructural, and financial resources. These resources are typically limited in low-income countries. Major intensive care units (ICUs) are mostly found in large hospitals in urban or metropolitan areas [2]. The most common admission criteria to these units are post-operative treatment, infectious diseases, trauma and obstetric complications [2,3].

A recent review highlighted the paucity of knowledge regarding critical care in the developing world [4]. Knowledge of the characteristics and outcomes of critically ill patients admitted to ICUs in low-income countries may help with the identification of priorities and the resources required for improvement of the care of critically ill patients in resource-limited regions of the world.

The aim of this study was to determine the admission diagnoses and outcome of patients admitted to the Mulago Hospital ICU from 2003 until 2009 and to highlight the country’s ICU bed capacity. It is hoped that the findings will be a useful addition to the increasing body of evidence highlighting the disparities between critical care in high- and low-income countries.

### Methods and results

This study was a retrospective audit. The study protocol was approved by the hospital Research and Ethics Committee. Medical charts were reviewed and anonymity was preserved for each case record.

The study intensive care unit is a 12-bed unit with the ability to ventilate only six patients at any one time. It provides level II ICU services to all kinds of critically ill patients. Level II care includes mechanical ventilation for longer than 24 h, and specific organ support like dialysis and inotropic infusions. The ICU can provide mechanical ventilation, post-operative care, intermittent haemodialysis, peritoneal dialysis, and basic neurocritical care. The ICU serves Mulago Hospital, which is a 1,500 bed national referral hospital, and Makerere University teaching hospital. The unit was started in the late 90s with a foreign donation and was initially run by a UK trained anaesthesiologist who has since retired. The ICU is currently staffed by three full-time ICU doctors (two internists and an anaesthesiologist) who have undergone further training in higher income countries, and 20 nurses. It receives technical support from the Department of Anaesthesia.

Apart from the study ICU, Mulago hospital also has a four-bed cardiac ICU, a four-bed coronary care unit (the heart institute is a semi-autonomous unit within the hospital that caters for paying patients and open-heart surgery patients), a six-bed paediatric high dependency unit, a new five-bed obstetric high dependency unit and a neonatal special care unit that can only provide nasal CPAP ventilation. No unit in the hospital can ventilate infants or neonates. Currently, the study ICU uses early warning score criteria to admit patients, together with a first come first served basis system, due to the limited number of beds. It is estimated that about ten critically ill patients are deprived of ICU admission daily, and as a result succumb to their illnesses. An ongoing study is being conducted in the hospital to identify missed opportunities for saving such patients.

The audit included all patients admitted to the study ICU from January 1, 2003, until December 31, 2009. No patient was excluded from the study. The following information was recorded for each study patient: basic demographic data (including age and gender), admission criterion, duration of stay in the ICU, and survival to ICU discharge. We also conducted a telephonic survey to establish the ICU bed capacity in the whole country.

### Results

Basic descriptive statistics were used to analyse demographics data and other study variables. Logistic regression analysis was used to determine the association between different durations of ICU stay and survival to discharge. P-values <0.05 were considered statistically significance. Data are presented as mean values, with standard deviations, unless otherwise indicated.

For the purposes of the telephonic survey, an ICU bed was defined as comprising a bed, a pulse oximeter, a mechanical ventilator, a suction machine and an anaesthesia provider in the vicinity. We determined that, based on our definition, there were 33 ICU beds in the whole country for a population of 33 million people (Table 1).

**Table 1:** Uganda’s functional ICU bed capacity

**Hospital**	**Status**	**Bed capacity**	**Anaesthesiologist/ICU trained physician available**
Mulago national referral hospital	Public University teaching	6	Yes
Mbarara university hospital	Public university teaching	4	Yes
Ishaka hospital	Private university teaching	2	Yes
Lacor hospital	Missionary	6	Yes
Jinja hospital	Public	N/A	No (unit not yet functional)
International hospital Kampala	Private	6	Yes
Case hospital	Private	3	Yes
Uganda heart institute	Private cardiac	6	Yes
Nakasero hospital	Private	3	Yes

ICU bed defined as comprising a bed, a mechanical ventilator, a pulse oximeter, a suction machine, and the presence of an anaesthesiologist.

During the study period, 1,774 patients were admitted to the study ICU (Table 2). The mean age of the study patients was 35.5 ears. The majority of the patients (56.5%) were male. Indigenous Ugandans accounted for the majority (92.8%) of the patients. Upcountry referrals constituted 42.9%, and the remaining patients were from within and around Kampala, the capital city of Uganda. The mean mortality rate over the 7-year period was 40.1% (n = 715) (Table 3). The highest mortality rate (44%) was observed in 2004; the lowest mortality rate (33.2%) was observed in 2005. Children (age <18 years) accounted for 11.6% of admissions, and their mortality rate was 40.1%, with paediatric post-operative admissions being higher than paediatric medical admissions.

**Table 2:** Demographic data and mortality of study patients

**Variable**	
**Age: Mean**	35.52
**Length of stay in a hospital unit: Median (IQR)**	3 (4-17)
**Sex**	**n (%)**
Male	1002 (56.5%)
Female	772 (43.5%)
**Origin**	**n (%)**
Kampala	989 (55.8)
Outside of Kampala	760 (42.9)
Not recorded	24 (1.4)
**Nationality**	**n (%)**
Local	1646 (92.8)
Foreigner	108 (6.1)
Not recorded	19 (1.1)
**Mortality by referring unit/specialty**	**n (%)**
Neurosurgery	177 (24.8)
Medical emergency	273 (38.2)
Surgical emergency	54 (7.6)
Post-operative	105 (14.7)
Obstetric	22 (3.1)
Paediatric post-operative	32 (4.5)
Paediatric medical	51 (7.1)
Not recorded	1 (0.1)
**Mortality by top 4 major working diagnoses**	**n (%)**
Sepsis	126 (49.0)
Head injury	147 (45.3)
Acute respiratory distress syndrome/acute lung injury	89 (41.8)
HIV/AIDS related	63 (35.6)

**Table 3:** Mortality rate by year and overall mortality rate over the 7 years

**Year**	**Outcome**		**Total n**
	**Died n (%)**	**Died n (%)**	
**2003**	145 (42.0)	200 (58.0)	345
**2004**	204 (44.0)	260 (56.0)	465
**2005**	63 (33.2)	127 (66.8)	190
**2006**	56 (35.7)	101 (64.3)	157
**2007**	68 (43.0)	90 (57.0)	158
**2008**	82 (36.8)	141 (63.2)	223
**2009**	97 (41.1)	139 (58.9)	236
**Total**	**715 (40.3)**	**1058 (59.7)**	**1774**

Sepsis, ARDS, traumatic head injury, and HIV/AIDS were the most frequent admission diagnoses during the study period (Table 2). Neurosurgical conditions accounted for the ICU admission diagnosis with the highest mortality.

**Figure 1:**
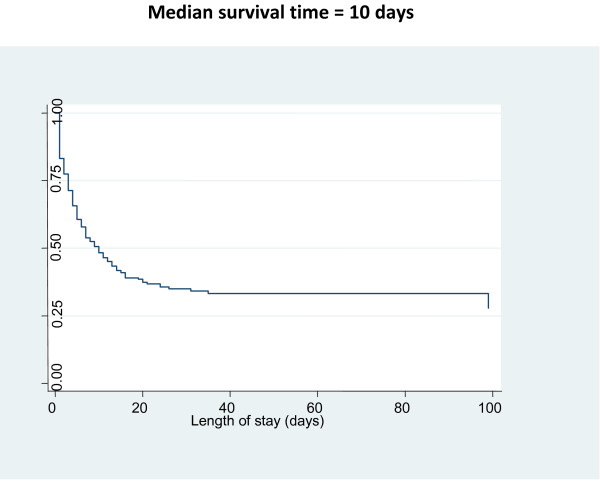
Survival curve length of stay and probability of survival.

Patients who stayed in the hospital for 6 to 10 days were three times more likely to survive compared with patients who stayed for 1 to 5 days. Patients who stayed for 11–20 days were at twice as likely to surviveas likely to die compared with patients who stayed for 1 to 5 days (Figure 1) (Table 4). Patients who stayed for >20 days were almost twice as likely to survive compared with patients staying for 1 to 5 days.

**Table 4:** Association between the length of stay in the ICU and patient outcomes

**Length of stay in Hospital unit (days)**	**Out come**	**OR**	**P-value**	**95% CI**
	**Died N (%)**			
1 – 5	571 (43.9)	1		
6 – 10	80 (34)	0.34	0.001	0.15 – 0.61
11 – 15	32 (32.3)	0.46	0.04	0.22 – 0.97
16 – 20	8 (28.6)	0.5	0.09	0.22 – 1.12
>20	10 (19.2)	0.6	0.34	0.20 – 1.74

### Discussion

In this retrospective audit, we aimed to determine admission patterns in our ICU during a 7-year observation period. We found that the two most common admission diagnoses were identical to those reported by ICUs located in other parts of the world [5-7]. The overall mortality rate of 40.1% is comparable to reports from other African country ICUs [5], but much higher than that reported by ICUs in high-income regions of the world (at between 10–20.9%) [6-8].

Head injuries were a common reason for ICU admission and associated with the highest mortality rates in this audit. This is not surprising, considering that the study ICU is a general ICU and does not have specialised neurocritical care resources (e.g. facilities to measure intracranial pressure or arterial blood gases). This is despite the ICU being served by four neurosurgeons; therefore the limitations are related to infrastructure rather than skills or personnel. It was difficult to determine what proportion of deaths was preventable because reliable data for this was not available.

A paper by Mock et al. estimated that improved trauma systems can avert between one and two million deaths a year in severely injured patients in low- and middle-income countries [9]. The lack of neurocritical monitoring equipment is coupled with the fact that Uganda does not have a functional emergency medical response system. This leads to inadequate transportation of trauma victims to health care facilities and delays in definitive care.

There are a limited number of ICU beds in Uganda as a whole - only one ICU bed for every one million Ugandans or 0.1 ICU beds/100,000 (Table 1). This compares poorly with South Africa (8.9/100,000), Sri Lanka (1.6/100,000), and the United States of America (20/100,000) [1]. This also explains the high number of referrals to Mulago hospital from upcountry centres. This limitation is further compounded by a well-documented dearth of anaesthesiologists- a critical human resource for intensive care units [10,11].

Adequate emergency care at a crash scene (e.g. airway management, positioning, oxygen and fluid resuscitation) is known to improve trauma outcome [12]. The high number of non-helmet wearing motorcycle riders in Uganda, and in Kampala in particular, also contributes to the high injury severity and mortality rate of neurotrauma observed in this study [13].

Sepsis was also a common cause of mortality, with mortality rates higher than those reported from industrialised countries [6-8]. Although our study data cannot explain the high mortality rates associated with sepsis, it is likely that insufficient early sepsis care may have contributed. Delayed presentation of sepsis patients to the hospital, and subsequently to the ICU, is common [14]. The paucity of resources to manage patients with sepsis (e.g. insufficient amounts of fluids, unavailability of intravenous broad-spectrum antibiotics and unavailability/unreliability of microbiological diagnostics) may have prevented adequate sepsis management at the study ICU. Recently, an expert group published guidelines to help resource poor settings manage critically ill patients with sepsis [15]. The recommendations have been well received in a number of resource limited countries.

The patient population included in this study is younger compared with patients admitted to ICUs in industrialised countries [16]. However, our findings are similar to those reported from surveys of critically ill patients treated in other African countries, where life expectancy is comparably low to that of Uganda [5]. Similarly, the mean length of ICU stay in this study resembled that in other parts of Africa. The finding that patients who stayed 6 to 15 days in the ICU experienced better survival to discharge than those treated for less than 5 days or longer than 2 weeks indicates that patients in the study ICU typically die early (within a few days) or relatively late (after 2 weeks). Early deaths can most likely be explained by the lack of trained staff and resources to provide adequate care for critically ill patients with a high disease severity (e.g. those with brain trauma, shock or sepsis).

Children accounted for 11% of all ICU admissions with a mortality rate of 40%. This is similar to other African country ICUs [17,18], but considerably higher than in industrialised countries [19]. The lack of ventilators and accompanying resources in the paediatric high dependency unit at the Mulago hospital is one of the main reasons why children are admitted to the study ICU. Although our study cannot prove a causal relationship, it is likely that delayed initiation of mechanical ventilation and aggressive resuscitation could explain the high death rate in the paediatric patients in the study population There was a higher mortality in the paediatric medical group than in the surgical group, and we hypothesise that this is because a lot of the post-operative patients were elective surgical patients who were admitted for observation. Most paediatric referrals were, and continue to be, children with acute respiratory failure who are transferred from the paediatric high dependency unit because they are in need of mechanical ventilation. The relatively younger population in LICs and the fact that respiratory illness is the leading cause of deaths in under-5-year olds in such countries [20], implies that more emphasis should be placed on strengthening paediatric critical care resources in LICs. Previous studies have suggested the need to estimate the cost effectiveness of critical care in this setting, given the relatively younger and economically active population. [1,2]

The fact that HIV is endemic in Uganda explains why HIV/AIDS was one of the most common reasons for admission in the study population. Due to the advent of easily accessible highly active anti-retroviral therapy, together with septrin prophylaxis, the incidence of HIV-related diseases (such as pulmonary infection with *Pneumocystis jiroveci*, which usually presents as acute respiratory failure) has markedly decreased [21,22]. In this survey, it was difficult to retrospectively determine from the medical records whether acute respiratory failure was due to infection or other causes. We could, however, determine that chronic obstructive pulmonary disease was a very rare cause of acute respiratory failure in our setting. Other rare HIV-related causes of ICU admission were viral encephalitis and liver failure.

Obstetric admissions in our study were largely due to perioperative cardiac arrest occurring as a consequence of peripartum haemorrhage, eclampsia and/or sepsis. Following the introduction of protocolised care for peripartum emergencies and the establishment of the obstetric high dependency unit (patient monitors and more intense nursing and protocols without mechanical ventilation) at the Mulago hospital, the number of obstetric critically ill patients admitted to the study ICU dropped substantially.

Limitations of this study include its retrospective nature with the consequence that it could not provide the same level of evidence as a prospective survey. Furthermore, due to the concise format of medical records, only limited data could be retrieved for this audit. For example, information on whether patients received mechanical ventilation; the volume of fluids; and drugs was not available. According to anecdotal evidence, 99% of all admissions are mechanically ventilated; however, the lack of data to support this precludes us stating this as a fact. Other ICU-relevant data would have allowed better description of the study population. More detailed data may have allowed for examination of other variables associated with mortality. It is hoped that advances in health information technology in low-income countries will result in improved reporting ability in the future.

### Conclusions

This is the largest study to date of critically ill patients in a low-income setting in sub-Saharan Africa. Our ICU study population is a young one and, even though we have limited data for comparison, high-income countries may have an older ICU population. We had a mortality rate of 40.1%, with sepsis, head injury, acute lung injury and HIV/AIDS the most common admission diagnoses. The mortality rate stayed the same over time, possibly because the admitting doctors stuck to their prognoses, and there are limitations in resources and a paucity of use of evidence-based practice. The fact that half the patients came from outside of the capital city is explained by the dearth of ICU beds in the country as a whole. Critical care remains a neglected area of health service delivery in this setting, with large numbers of patients with potentially treatable conditions not having access to such services. Further research needs to be carried out in ICUs in other resource limited settings, including a prospective study to estimate the resources required to design resource appropriate units in such settings and the impact on morbidity and mortality, especially for the most common conditions.

## Abbreviations

HIV/AIDS: Human Immunodeficiency Virus/Acquired Immunodeficiency Syndrome; ICU: Intensive care unit; LIC: Low-income country; HIC: High-income country.

## Competing interests

The authors declare that they have no competing interests and received no funding for this study.

## Authors’ contributions

AK conceived the study, and participated in the study design, data and statistical analysis, and drafting the manuscript. MD participated in the study design, data analysis and drafting the manuscript. JN participated in data collection, data analysis and drafting the manuscript. All authors approved the final manuscript.
